# Targeting HER2-positive breast cancer cells by a combination of dasatinib and BMS-202: Insight into the molecular pathways

**DOI:** 10.1186/s12935-023-03195-z

**Published:** 2024-03-02

**Authors:** Hadeel Kheraldine, Ishita Gupta, Farhan Sachal Cyprian, Semir Vranic, Halema F. Al-Farsi, Maysaloun Merhi, Said Dermime, Ala-Eddin Al Moustafa

**Affiliations:** 1 College of Medicine, QU Health, Qatar University, P. O. Box 2713, Doha, Qatar; 2 Biomedical Research Centre, Qatar University, P. O. Box 2713, Doha, Qatar; 3 National Center for Cancer Care and Research, Hamad Medical Corporation, Doha, Qatar; 4 Translational Cancer Research Facility, Interim Translational Research Institute, Hamad Medical Corporation, Doha, Qatar; 5 Oncology Department, Faculty of Medicine, McGill University, Montreal, QC Canada; 6 Present Address: Sidra Medicine, Doha, Qatar

**Keywords:** Dasatinib, PD-1/PD-L1, HER2-positive breast cancer, EMT, Invasion

## Abstract

**Background:**

Recent investigations have reported the benefits of using a tyrosine kinase inhibitor, dasatinib (DA), as well as programmed death-ligand 1 (PD-L1) inhibitors in the management of several solid tumors, including breast cancer. Nevertheless, the outcome of the combination of these inhibitors on HER2-positive breast cancer is not explored yet.

**Methods:**

Herein, we investigated the impact of DA and PD-L1 inhibitor (BMS-202) combination on HER2-positive breast cancer cell lines, SKBR3 and ZR75.

**Results:**

Our data reveal that the combination significantly inhibits cell viability of both cancer cell lines as compared to monotreatment. Moreover, the combination inhibits epithelial-mesenchymal transition (EMT) progression and reduces cancer cell invasion by restoring E-cadherin and β-catenin expressions and loss of vimentin, major biomarkers of EMT. Additionally, the combination reduces the colony formation of both cell lines in comparison with their matched control. Also, the combination considerably inhibits the angiogenesis of the chorioallantoic membrane model compared with monotreatment. Molecular pathway analysis of treated cells shows that this combination blocks HER2, AKT, β-catenin, and JNK1/2/3 activities.

**Conclusion:**

Our findings implicate that a combination of DA and BMS-202 could have a significant impact on the management of HER2-positive breast cancer.

## Background

Breast cancer is the most prevalent form of cancer amongst women worldwide, with high incidence and mortality rates [1]. Perou et al. [2] first classified breast cancer based on gene expression profiling using hierarchical cluster analysis into four molecular subtypes; Luminal (A and B), HER2-positive, basal-like, and normal-like [2]. Of all subtypes, HER2-positive overexpresses the human epidermal growth factor receptor type 2 (EGFR2, ErbB2, or HER2) and comprises 15–25% of total breast cancer cases [3, 4]. Current therapeutic regimens for HER2-positive breast cancer include monoclonal antibodies, tyrosine kinase inhibitors, and cytotoxic chemotherapy, in addition to hormonal therapy for steroid receptor-positive cases, such as trastuzumab, lapatinib, pertuzumab, neratinib and ado-trastuzumab emtansine [5–9]. However, these chemo/immunotherapies pose several limitations, including resistance, early relapse, poor prognosis, and higher recurrence rate in addition to complications of toxicity [10–12].

Dasatinib (DA), an oral tyrosine kinase inhibitor, is used for the treatment of chronic myeloid leukemia and Philadelphia chromosome-positive acute lymphoblastic leukemia [13]; it is known to successfully block Src and Src-family kinases (SFKs) [14]. A functional relationship was observed between Src and HER2, as increased Src activity was correlated with increased metastasis potential in HER2-positive breast cancer [15–18]. Inhibiting Src activity in-vitro and in-vivo can reduce HER2-positive breast cancer cell invasiveness and metastasis [19, 20]. A phase 1 study in solid tumors demonstrated the efficacy and safety of DA monotherapy [21]. Furthermore, in breast cancer, one phase 2 study in the triple-negative subtype showed that DA was effective [22], while another phase 2 study showed that DA monotherapy was more effective in patients with hormone-receptor-positive breast cancer in comparison with HER2-positive breast cancer [23]. On the other hand, the immune checkpoint, programmed cell-death ligand-1 (PD-L1) is significantly expressed in triple-negative followed by the HER2-positive breast cancer subtypes [24–28]; thus, indicating that HER2-positive breast cancer is immunogenic [29]. Immune checkpoint inhibitors include anti-PD-1 or PD-L1 agents (pembrolizumab, nivolumab, avelumab, and durvalumab) and are involved in inhibiting breast cancer, especially the triple-negative subtype when administered as a monotherapy or in combination [30, 31]. In this regard, a phase III trial (IMPassion130) involved a combination of immunotherapy (atezolizumab) with chemotherapy (nab-paclitaxel) that improved the outcome of patients in the PD-L1 positive group with triple-negative metastatic breast cancer [32]. However, no positive response was observed in HER2-positive breast cancer in the JAVELIN phase I study when it was administered with a single-agent avelumab [33]. Moreover, another phase Ib/II study (PANACEA (IBCSG 45 − 13/BIG 4–13/KEYNOTE-014)) analyzed the synergistic effect of pembrolizumab and trastuzumab in patients with trastuzumab-resistant HER2-positive breast cancer; although PD-L1 positive patients initially responded, they ultimately developed resistant disease [34]. Since resistance to different types of HER2 drugs is still a major challenge in the management of HER2-positive breast cancer, the potency of new targets, including PD-1/PD-L1 inhibitors that can be used with potential anti-HER2 drugs needs to be explored further.

On the other hand, in HER2-positive breast cancer, phase II (GEICAM/2010-04) trials of DA in combination with trastuzumab and paclitaxel as a first-line treatment showed good efficacy [35]. Previous studies in triple-negative breast cancer have investigated the efficacy and safety of DA [22, 36, 37] and PD-1/PD-L1-inhibitor [30, 38–40]; however, there are no studies on their combined effect in HER2-positive breast cancer. Thus, we herein explore for the first time the individual and combined effects of DA and PD-L1 inhibitor (BMS-202); a small molecule that blocks the interaction between PD-1 and PD-L1, on selected parameters in two HER2-positive human breast cancer cell lines. In addition, the outcome of these inhibitors together on angiogenesis has been examined using the chorioallantoic membrane (CAM) model.

## Methods

### Cell culture

HER2-positive breast cancer cell lines (SKBR3 and ZR75) were obtained from the American Type Tissue Culture (ATCC) (Rockville, MD, USA) and used to investigate the anticancer effects of DA and BMS-202. Cells were grown in complete cell culture medium Gibco® RPMI-1640 (Life Technologies, Burlington, ON, Canada) supplemented with 5% fetal bovine serum (FBS; Invitrogen, Life Technologies) and 1% PenStrep antibiotic (Invitrogen, Life Technologies). Human normal mammary epithelial cells immortalized by the E6/E7 gene of HPV type 16 (HNME-E6/E7) [41] were used as a control. These cells were maintained in Gibco® Keratinocyte-SFM (1X) medium (Thermo Fisher Scientific, Mississauga, ON, Canada) supplemented with 1% PenStrep antibiotic (Thermo Fisher Scientific, Mississauga, ON, Canada). Cells were kept at 37 °C with a 5% CO_2_ humidified atmosphere. All used cells were negative for mycoplasma contamination. Cells were tested for mycoplasma contamination and were negative.

### Fluorescence-activated cell sorting (FACS)

SKBR3 and ZR75 cells were harvested, washed with PBS, and stained with PE/Cyanine7 anti-human CD274 (B7-H1, PD-L1) antibody (Biolegend: 329,717). Afterward, cells were washed with FACS buffer and PD-L1 expression was explored using CytoFLEX (Beckman Coulter, USA). The data were processed by FlowJo V10 software.

### Preparation of treatments

Dasatinib; a tyrosine kinase inhibitor (Src inhibitor) (ab142050, Abcam, Cambridge, MA, USA), and BMS-202 (ab231311, Abcam, Cambridge, MA, USA); a small molecule inhibitor of PD-L1 were prepared in DMSO in a concentration of 10 mg/ml and kept as aliquots in -20 °C.

### Cell viability assay

SKBR3 and ZR75 cells were seeded in 96-well plates (Thermo Fisher Scientific, Mississauga, ON, Canada) in a density of 1 × 10^4^ cells/well. Cells were treated with different concentrations of DA (1, 5, 7, 10, 15, and 20 µM) and BMS-202 (1, 5, 7, 10, 15, 20, and 30 µM) for 48 h. A combined treatment of DA and BMS-202 was prepared at various concentrations (1, 2, 3, 5, 7, 10, and 20 µM) and used on both cell lines for 48 h. The concentration of DMSO did not exceed 0.1%. Control wells received 100 µl of media. According to the manufacturer’s protocol, cell viability inhibition was determined after 48 h of treatment using Alamar Blue Cell viability reagent (Invitrogen, Thermo Fisher Scientific). After 4 hours of incubation with the dye, the shifts in fluorescence were recorded at a wavelength of 560 nm (excitation) and 600 nm (emission) using the Infinite m200 PRO fluorescent microplate reader (TECAN, Männedorf, Switzerland). Relative cell viability was determined based on the fluorescence of drug-treated cells compared with that of control cells.

### Cell invasion assay

Cell invasion assay was carried out in 24-wells BioCoat™ Matrigel® Invasion Chambers (Corning, USA) with 8.0 μm PET Membrane as per the manufacturer’s protocol. Cells were seeded (5 × 10^4^ cells/well) in the upper insert-well and maintained in serum-free medium with/without treatment, while in each base-well complete medium with 10% FBS was added and then incubated at 37 °C. After 24-hour incubation, non-invasive cells in the upper insert were removed with a cotton swab. In contrast, invasive cells were fixed with 4% formaldehyde, permeabilized with methanol, and washed, followed by staining with 5% crystal violet dissolved in methanol. Cells were then visualized, and quantification was carried out under the Leica DMi1 inverted microscope (Leica Microsystems, Wetzlar, Germany) in five predetermined fields, as previously described [41]. The percentage inhibition of invasive cells was calculated for untreated cells.

### Soft agar colony formation assay

Soft agar assay was performed to evaluate cancer cells’ ability to form colonies before and post-treatment in agar. A total of 5 × 10^3^ cells of SKBR3 and ZR75 were placed in their medium containing 0.3% agar with/without treatment(s) of DA and BMS-202 (treated and control cells, respectively) and plated in a 6-well plate covered with a layer of 0.4% agar prepared in RPMI-1640 medium. Colony formation was monitored every 7 days for three weeks, followed by counting colonies in each well using the Leica inverted microscope (Leica Microsystems, Wetzlar, Germany).

### Immunofluorescence

SKBR3 and ZR75 cells were grown on coverslips and stained for immunofluorescence. Briefly, cells were treated with a combination of DA and BMS-202 for 48 h. Cells were washed with PBS and fixed with 4% of formaldehyde, followed by permeabilization using 0.2% of triton X-100. Cells were then washed and blocked with a 10% FBS blocker. Then, they were incubated with the primary antibody of E-cadherin and β-catenin (Abcam, Cambridge, MA, USA) overnight in a humidified chamber. On the next day, cells were washed and incubated with the corresponding secondary antibody in the dark. Afterward, DAPI staining with 300 ng/ml of DAPI solution (Abcam, Cambridge, MA, USA) was performed in the dark, then cells were mounted using Jelly mount water-based mounting media (DDk Italia, #04-108), and fluorescence was visualized by fluorescence microscope. Untreated cells served as a control.

### Western blotting

Alterations in the protein expression levels were analyzed by Western blotting as previously described by our group [42]. In brief, SKBR3 and ZR75 cells (2 × 10^6^ cells) were seeded and treated with DA and BMS-202, individually and in combination for 48 h. Cell lysates were collected, and equal amounts of protein (30 µg) were resolved on 10% SDS PAGE gels and electroblotted onto PVDF membranes, then probed with the following primary antibodies: anti-rabbit Src family (phospho Y418) (Abcam: ab40660), anti-mouse ErbB2 (Abcam: ab16901), anti-rabbit phosphorylated ErbB2 (Abcam: ab53290), anti-mouse E-cadherin (Cell Signaling: 14,472 S), anti-rabbit vimentin (Cell Signaling: 46,173 S), anti-rabbit β-catenin (Cell Signaling: 8480 S), anti-rabbit phosphorylated β-catenin (Cell Signaling: 4176 S), anti-rabbit AKT (Cell Signaling: 9272 S), anti-rabbit phosphorylated AKT (Cell Signaling: 4060 S), anti-rabbit JNK1/2/3 (Abcam: ab179461). Anti-rabbit GAPDH (Cell Signaling: 8480 S) was used to ensure equal loading of protein samples.

Immunoreactivity was analyzed using the ECL Western blotting substrate (Pierce Biotechnology, Rockford, IL, USA), as described by the manufacturer, and blots were imaged using the iBrightTM CL1000 imaging system (Thermo Fisher Scientific, Waltham, MA, USA). Quantification was done using ImageJ software as previously described by our group [43].

### Angiogenesis assay

The chorioallantoic membrane (CAM) of the chicken embryos was treated on day five of incubation to evaluate the outcome of 1 µM of DA and BMS-202, individually and together, on the vascular development of the CAM. The prepared drug was placed on a circular glass coverslip for 48 h, as previously performed by our group [44–47]. DMSO-treated embryos were used as controls. Post 48 h of treatment, we examined the vascular development of the CAM under a stereomicroscope, and images were captured and analyzed using AngioTool software version 0.6a [48].

### Statistical analysis

Data are presented as an average of mean ± SEM (standard error of the mean). Each experiment was repeated at least three times (n = 3). One-way ANOVA followed by Tukey’s posthoc test was used to compare the difference between treated and untreated cells. The data were analyzed using Microsoft Excel and GraphPad Prism software (version 8.4.3), and differences with *p* < 0.05 were considered statistically significant.

## Results

**Fig. 1:**
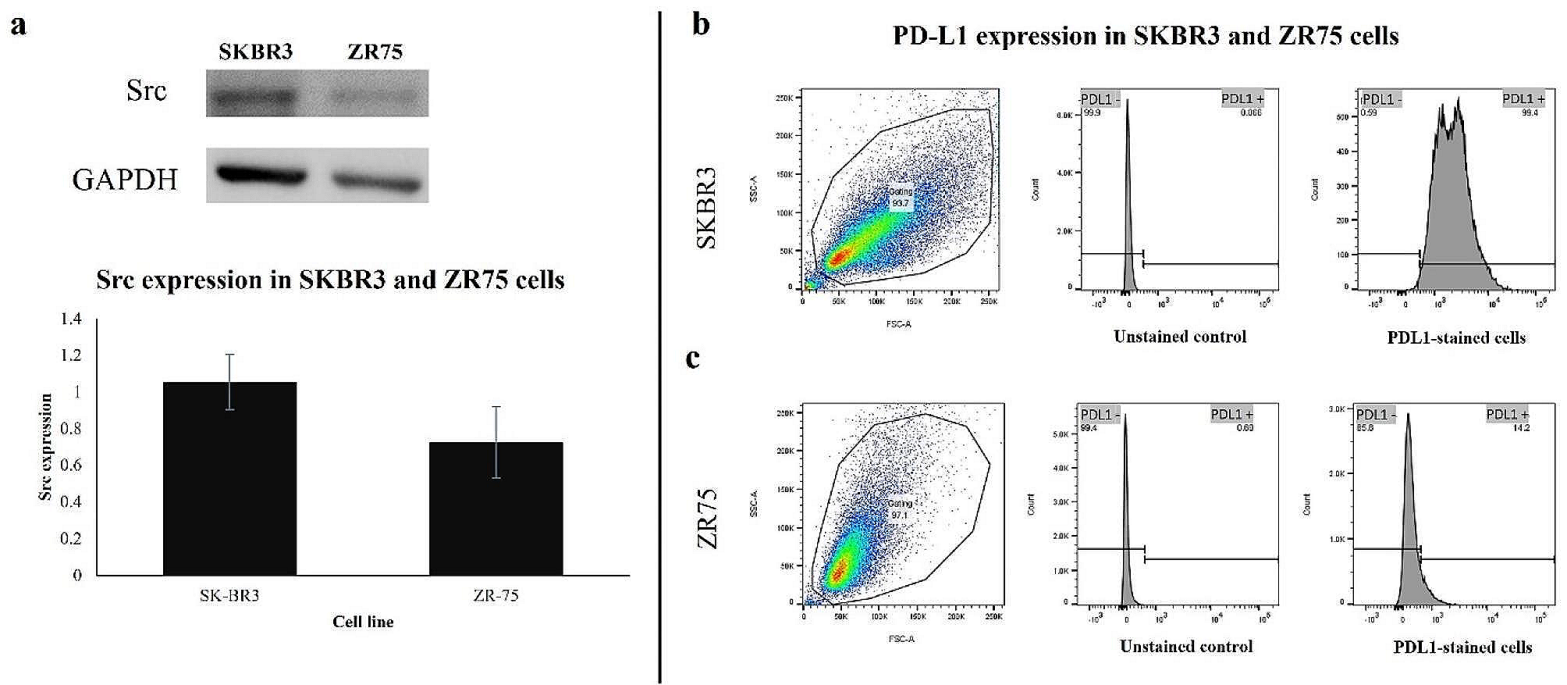
Both HER2-positive breast cancer cell lines, SKBR3 and ZR75, express drug targets: Src and PD-L1. (**a)** The expression of Src in SKBR3 and ZR75 cells is shown by Western blot. Data are expressed as src expression ± SEM. (**b)** PD-L1 expression in SKBR3 and (**c**) ZR75 cells shown by FACS analysis

The impact of DA and BMS-202 was explored in HER2-positive breast cancer cell lines, SKBR3 and ZR75. HNME-E6/E7 cells, human normal mammary epithelial cells immortalized by E6/E7 of HPV type 16 [41] were used as control. We first examined whether the HER2-positive breast cancer cell lines (SKBR3 and ZR75) express our drug targets (Src and PD-L1). As shown in Fig. 1a, both HER2-positive cancer cells express Src, with a higher expression seen in SKBR3 compared to ZR75. In addition, FACS analysis of cell surface proteins revealed that 99.4% of SKBR3 cells express PD-L1 ligand compared to 14.2% of ZR75 cells (Fig. 1b and c). Thus, we proceeded with the treatment and the following experiments.

**Fig. 2:**
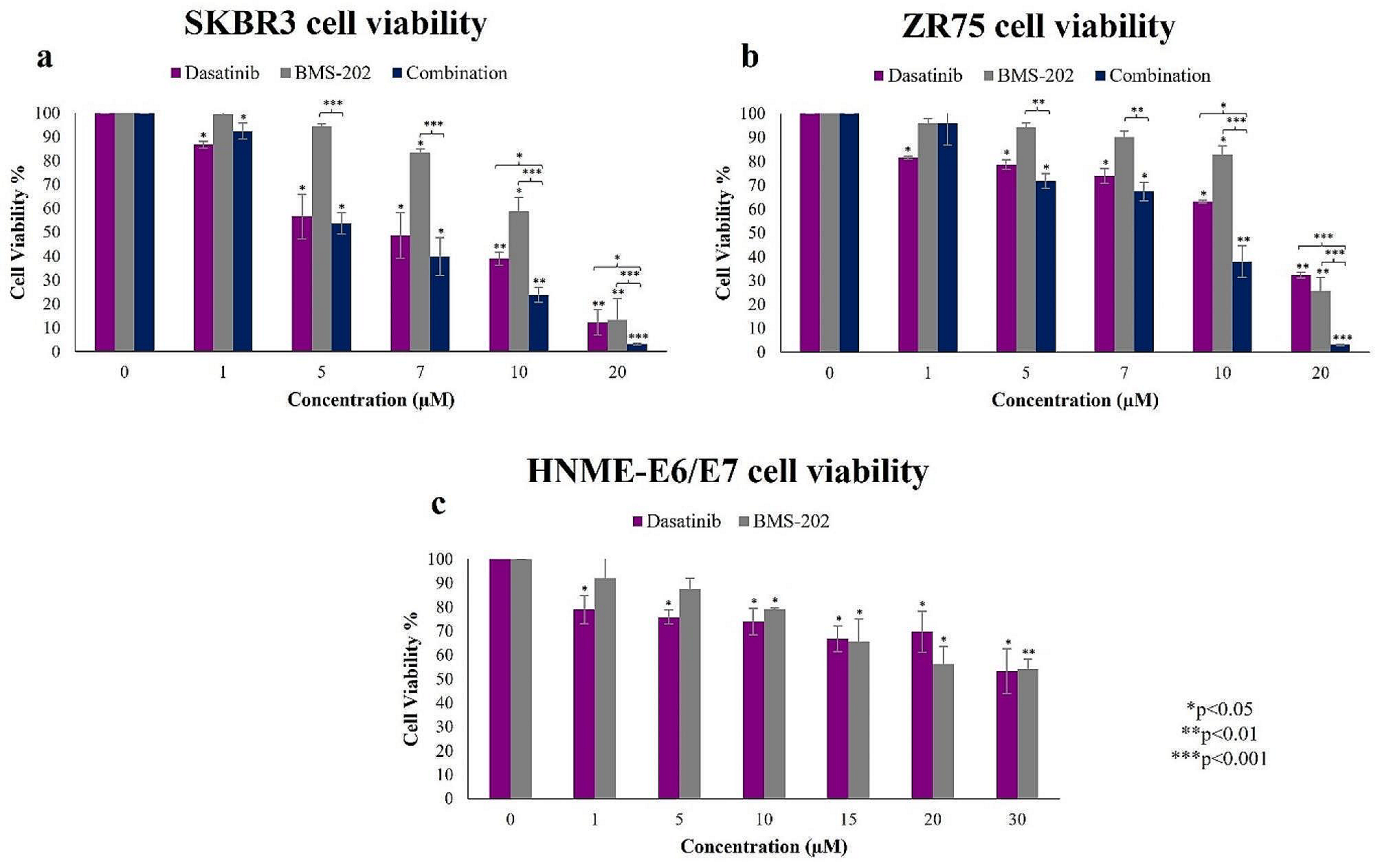
SKBR3, ZR75, and HNME-E6/E7 cell viability. The effects of different concentrations of dasatinib (DA) (0,1,5,7,10,20 µM), BMS-202 (0,1,5,7,10,20 µM), and combination therapy of both on cell viability of HER2-positive breast cancer cell lines (**a**) SKBR3 and (**b**) ZR75. (**c**) The effects of different concentrations of a combination of DA (0,1,5,10,15,20,30 µM) and BMS-202 (0,1,5,10,15,20,30 µM) on cell viability of human immortalized mammary epithelial (HNME-E6/E7) cells. Cells were treated for 48 h. Data indicate an inverse relationship between concentrations of DA and BMS-202, individually and in combination on cell viability in both cancer cell lines, in comparison to HNME-E6/E7 cells. Data are expressed as a percentage of growth ± SEM

Our data revealed that both inhibitors reduced the viability of SKBR3 and ZR75 cells significantly in a dose-dependent fashion (Fig. 2). Out of the various concentrations that were studied, from 1 to 20 µM range, the IC_50_ of DA was found to be 8.58 ± 0.08 µM and 13.89 ± 0.14 µM in SKBR3 and ZR75, respectively (Fig. 2a); While the IC_50_ of BMS-202 had a higher value of 12.84 ± 1.09 µM and 15.14 ± 0.46 µM, in SKBR3 and ZR75 cell lines, correspondingly (Fig. 2b). In combination treatment, a more significant decrease in cell viability is observed at 5 µM of DA and 5 µM of BMS-202 in both SKBR3 and ZR75 cell lines (Fig. 2a and b). Moreover, HNME-E6/E7 cells treated with DA showed some reduction in cell viability as well, but not as pronounced as in cancer cells. For instance, cell viability with a dose of 10 µM of DA is ~ 80% in HNME-E6/E7 cells compared to ~ 40% and ~ 60% in SKBR3 and ZR75, respectively. While HNME-E6/E7 cells treated with BMS-202 did not show a significant reduction in cell viability except at higher concentrations, starting from 10 µM (Fig. 2c).

After obtaining the IC50 values of DA and BMS-202 monotreatment, we calculated the combination index using the Chou-Talalay method [49] as per the below equation:

$$\left(\text{C}\text{I}\right) = \left(\text{D}\right)1 /\left(\text{D}\right)1 + \left(\text{D}\right)2/\left(\text{D}\right)2$$

- CI: combination index.
- (Dχ)1 and (Dχ)2: concentrations of each drug alone to exert χ% effect.
- (D)1 and (D)2: concentrations of drugs in combination to elicit the same effect.
- CI < 1, = 1 and > 1 indicated synergism, additivity and antagonism, respectively.

Based on our calculations, in SKBR3 cells, CI (SK) = 1.002295602 indicating an additive effect. While, in ZR75 cells, CI (ZR) = 0.826886187, indicating a synergistic effect.

**Fig. 3:**
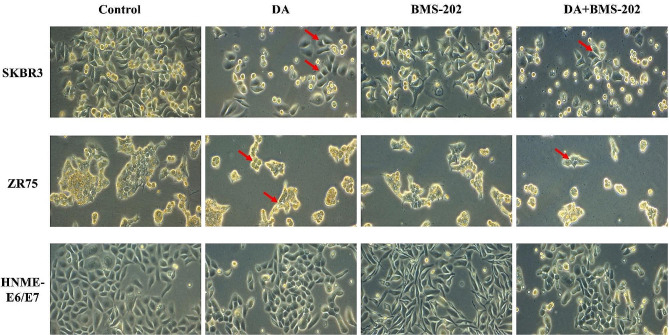
Effect of DA (5 µM) and BMS-202 (5 µM) on cell morphology of SKBR3, ZR75, and HNME-E6/E7 for 48 h. In controls, SKBR3 and ZR75 cells have round or polygonal morphology and form clusters. Upon treatment, we notice smaller clusters and cell death. The arrows indicate epithelial morphology with clear cell-cell adhesion. While DA and BMS-202 slightly affect the cell morphology of HNME-E6/E7 cells (images a and b at ×20 magnification)

Next, we investigated cell morphology alterations and EMT progression, in SKBR3 and ZR75 cells, in addition to HNME-E6/E7 cells upon treatment with 5 µM of DA and BMS-202, individually and combined. In the absence of treatment, SKBR3 and ZR75 cells displayed a round morphology and disorganized multilayered cells. In contrast, and as indicated in Fig. 3, treatment for 48 h with DA alone led to a phenotypic conversion from round cells to an “epithelial-like” phenotype. Clearly, cells became more flattened in appearance and showed an increase in cell-cell adhesion in comparison with untreated cells (Fig. 3). Interestingly, the impact of the combination treatment of DA and BMS-202 is far more significant than individual treatment concerning cell morphology and cell-cell contact in addition to the number of cells (Fig. 3). However, under DA and BMS-202, morphological changes induced in HNME-E6/E7 cells are less significant than cancer cells, as shown in Fig. 3.

**Fig. 4:**
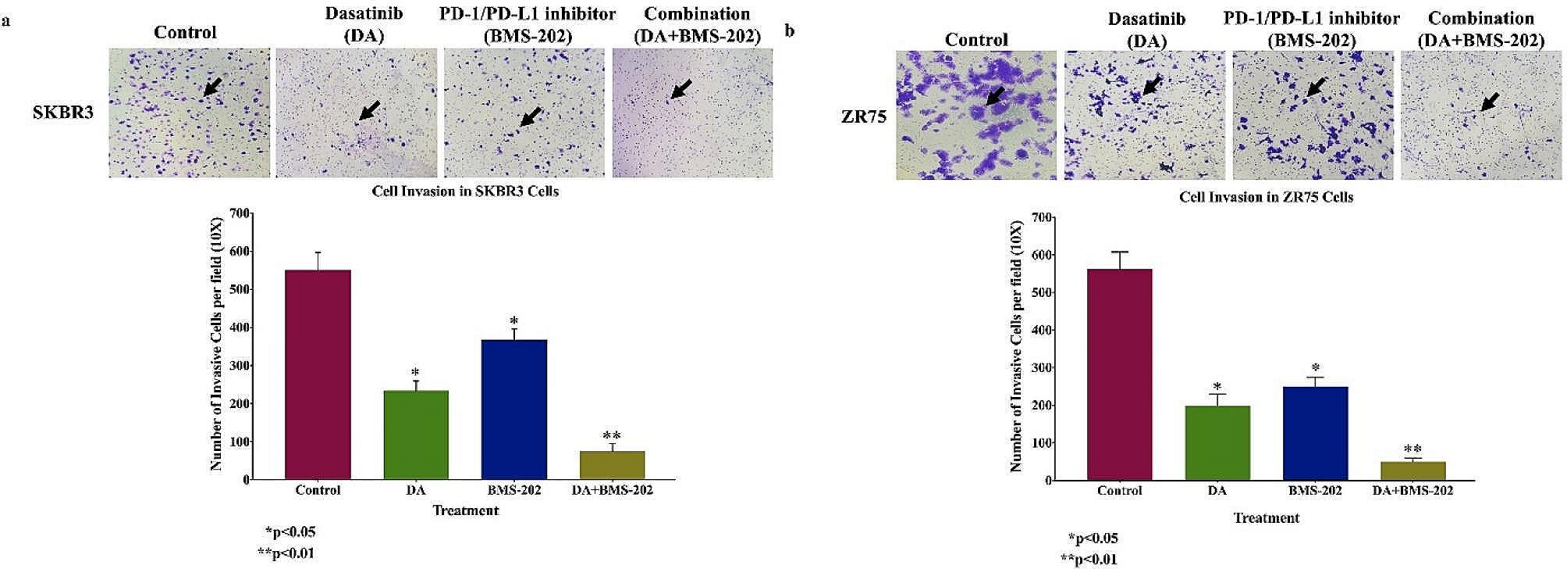
Effects of DA (5 µM) and BMS-202 (5 µM) on cell invasion of human HER2-positive breast cancer cell lines, SKBR3 and ZR75 for 48 h. DA and BMS-202 inhibit the cell invasion ability of (**a**) SKBR3 and (**b**) ZR75 cell lines using Matrigel® Invasion Chambers. We note that cancer cells treated for 48 h with DA and BMS-202 show significant inhibition of cell invasion in both cell lines compared with their matched control. Data are quantified by normalizing the number of invasive cells by their total number. Data are expressed as the number of invasive cells per field ± SEM

Subsequently, we analyzed the anti-invasion ability of DA and BMS-202, alone and in combination in both cell lines, SKBR3 and ZR75, using Matrigel® Invasion Chambers. The combined treatment of DA and BMS-202 showed a dramatic decrease in the number of invasive cells upon treatment compared to each treatment individually (Fig. 4). This suggests that both DA and BMS-202 can considerably downgrade cell invasion and consequently cancer progression of HER2-positive breast cancer.

**Fig. 5:**
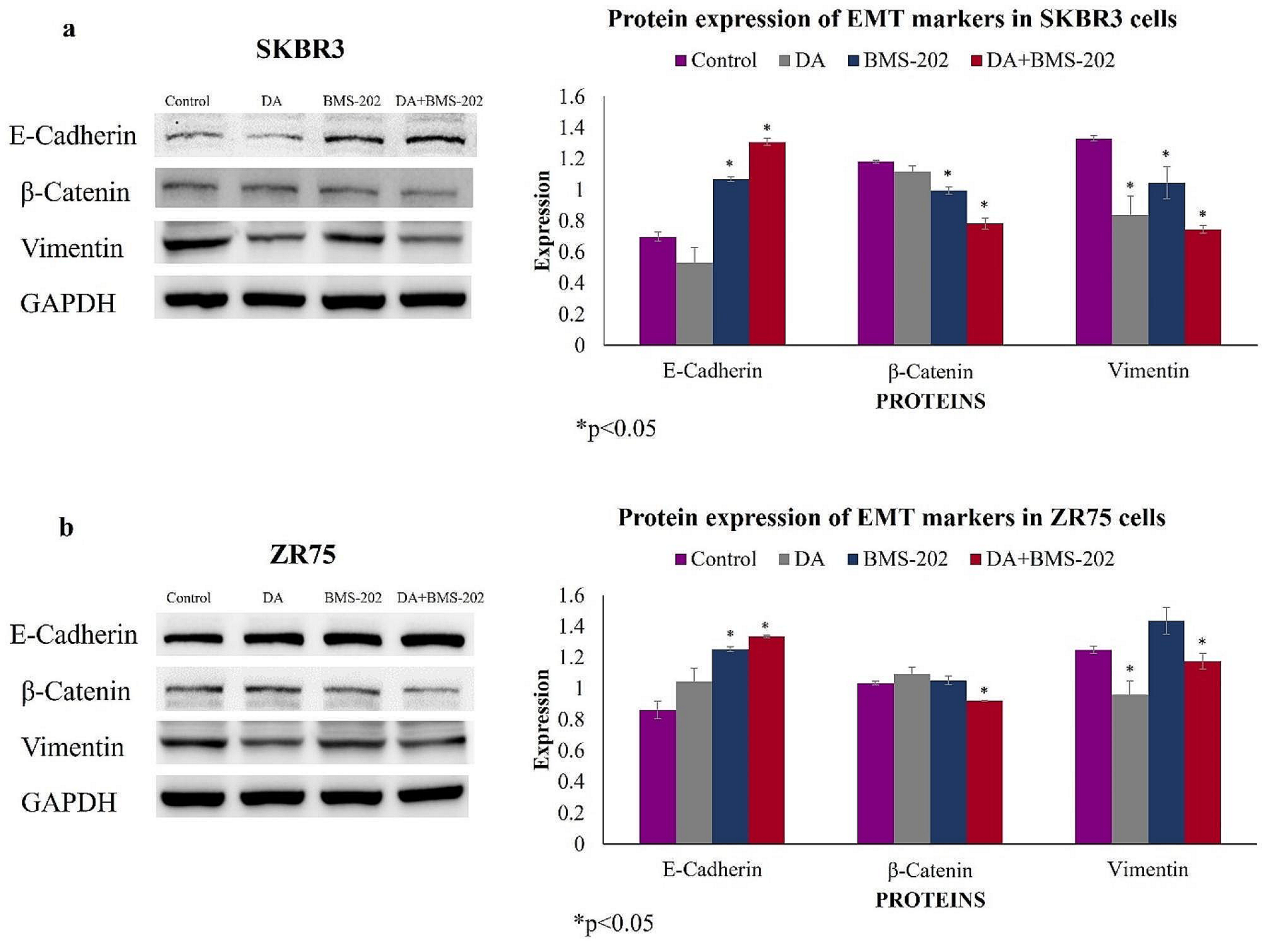
Western blot analysis of E-cadherin, β-catenin, and vimentin expression in (**a**) SKBR3 and (**b**) ZR75 cells under the effect of DA (5 µM) and BMS-202 (5 µM) for 48 h. It is evident that treatment with both DA and BMS-202 upregulates E-cadherin in both cell lines while downregulating β-catenin and vimentin expression in comparison with DA and BMS-202 treatment alone and their matched control. GAPDH was used as a loading control. Cells were treated with DA and BMS-202 for 48 h, as illustrated in the materials and methods section. Data are expressed as protein expression ± SEM

Based on the above data, we explored the expression patterns of key marker genes of EMT and cancer invasion, E-cadherin, β-catenin, and vimentin; our data pointed out that combination treatment (DA and BMS-202) exerted a significant effect in comparison to individual treatment. For instance, our Western blot analysis demonstrates that the combination of DA and BMS-202 enhances E-cadherin expression in SKBR3 and ZR75 cell lines. In contrast, β-catenin and vimentin expression was decreased compared to individual treatment and untreated control cells (Fig. 5).

**Fig. 6:**
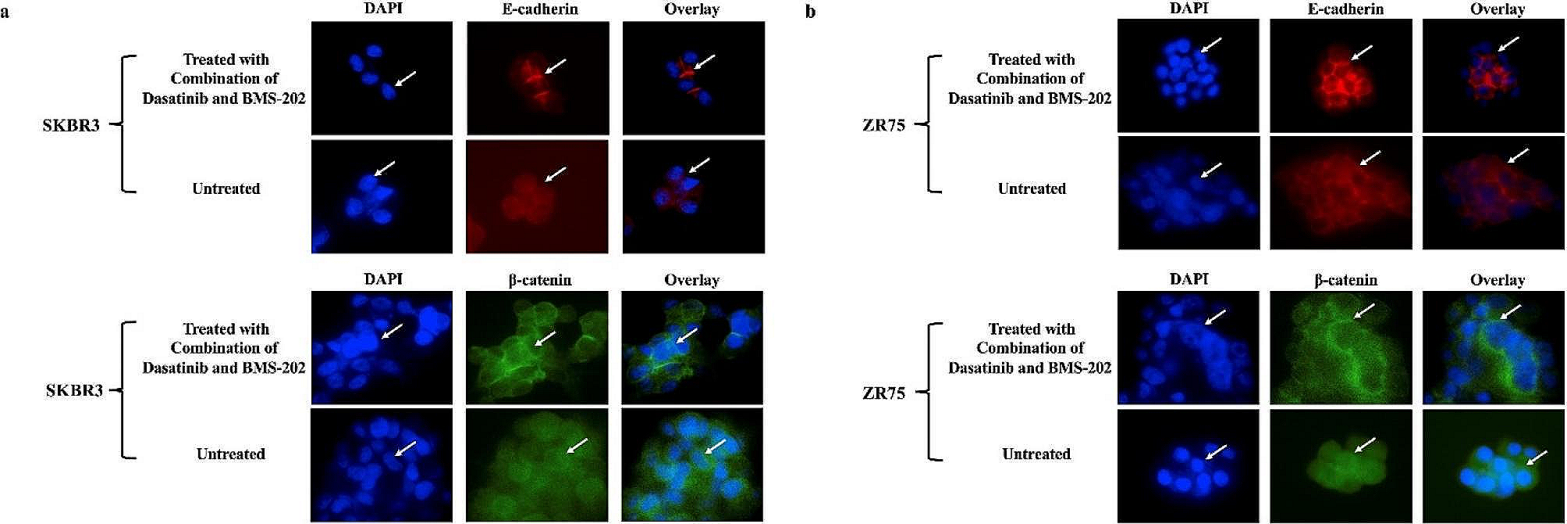
Immunofluorescence analysis of E-cadherin and β -catenin expression patterns of (**a**) SKBR3 and (**b**) ZR75 cells. Treatment with DA (5 µM) and BMS-202 (5 µM) for 48 h enhances the expression of E-cadherin in both cancer cell lines and promotes the translocation of E-cadherin and β-catenin from the cytoplasm to the cell membrane and its undercoat, respectively, in comparison with DA and BMS-202 alone as well as untreated cells. Cells were treated as described in the materials and methods section

Following the previous results, our immunofluorescence analysis reveals that treatment with a combination of DA and BMS-202 regulates E-cadherin and β-catenin expression patterns (Fig. 6). More specifically, the dual treatment promotes the translocation of E-cadherin and β-catenin from the cytoplasm to the cytoplasmic membrane and its undercoat, respectively (Fig. 6). However, in untreated cells, E-cadherin and β-catenin are equally distributed in the cytoplasm (Fig. 6). These data indicate that DA and BMS-202 combined, prevent EMT progression of both cell lines, SKBR3 and ZR75, via the restoration of the E-cadherin/β-catenin complex.

**Fig. 7:**
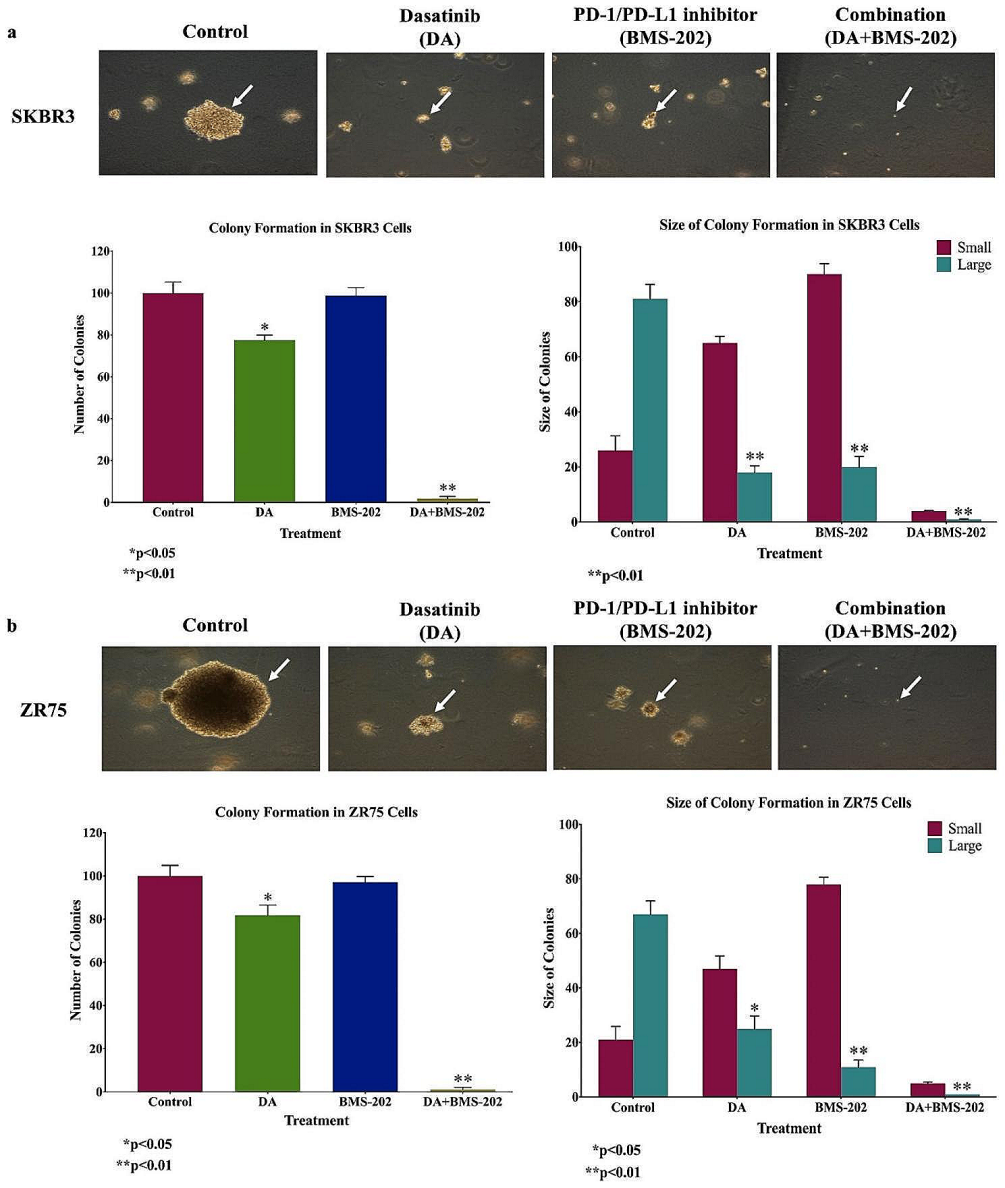
The outcome of DA (5 µM) and BMS-202 (5 µM) on colony formation of (**a**) SKBR3 and (**b**) ZR75 cell lines in soft agar after 21 days. DA and BMS-202 inhibit colony formation of SKBR3 and ZR75 in comparison with their matched control cells. Colonies were counted manually and expressed as a percentage of treatment relative to the control (Mean ± SEM)

On the other hand, we examined the effect of DA and BMS-202, alone and in combination, on the colony formation of cancer cells in soft agar. Our data show a significant decrease in colony number and size in cells treated with DA and BMS-202 individually (*p* < 0.001) in comparison with their controls (Fig. 7). Interestingly, cells treated with the two drugs combined did not form any colonies in both cell lines (*p* < 0.001) (Fig. 7). These data indicate that treatment with DA and BMS-202 together significantly suppress colony formation of HER2-positive breast cancer and probably tumor growth *in-vivo.*

**Fig. 8:**
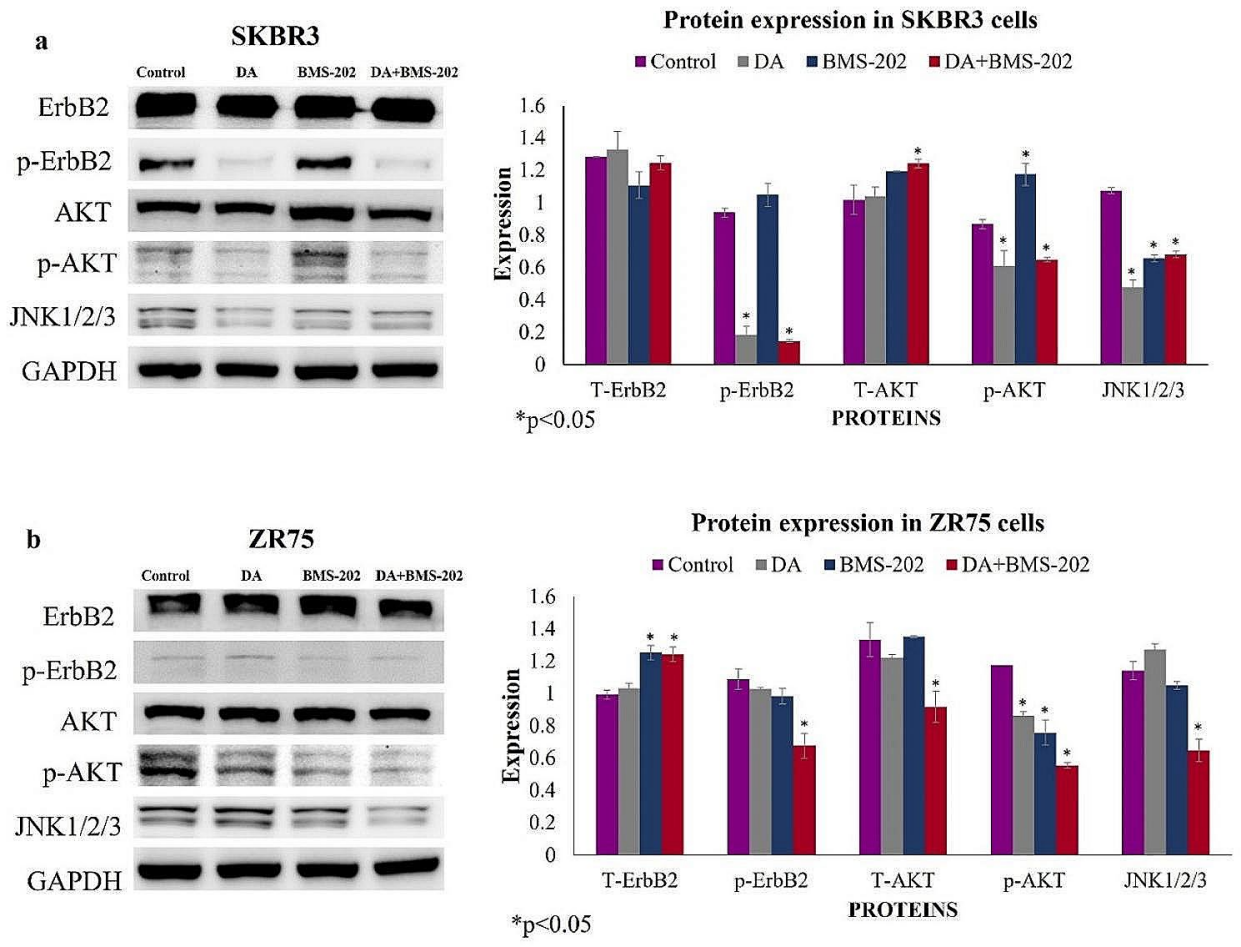
Molecular pathways analysis of DA (5 µM) and BMS-202 (5 µM) in (**a**) SKBR3 and (**b**) ZR75 cell lines after 48 h of treatment. DA and BMS-202 inhibitors together reduce the phosphorylation of ErbB2 and AKT, as well as the expression of JNK1/2/3 in both cell lines in comparison with those treated with DA and BMS-202 individually and control cells. GAPDH was used as a control for the amount of protein in this assay. Data are expressed as protein expression ± SEM

Vis-à-vis the underlying molecular pathways of these drugs on cell viability, EMT progression, cell invasion, and colony formation of HER2-positive breast cancer cells, we assumed that HER2 inactivation in addition to PI3K/AKT and c-Jun N-terminal kinase (JNK) pathways could have major roles in regulating these events [50–54]; therefore, the expression patterns of HER2, AKT, and JNK1/2/3 were explored. As seen in Fig. 8, the expression and phosphorylation of HER2 (ErbB2) were downregulated after treatment with DA alone and the drug combination; nevertheless, BMS-202 alone did not affect HER2 expression and activation. Furthermore, the expression patterns of AKT (phosphorylated) and JNK1/2/3 expression were reduced after combination treatment compared to the controls.

**Fig. 9:**
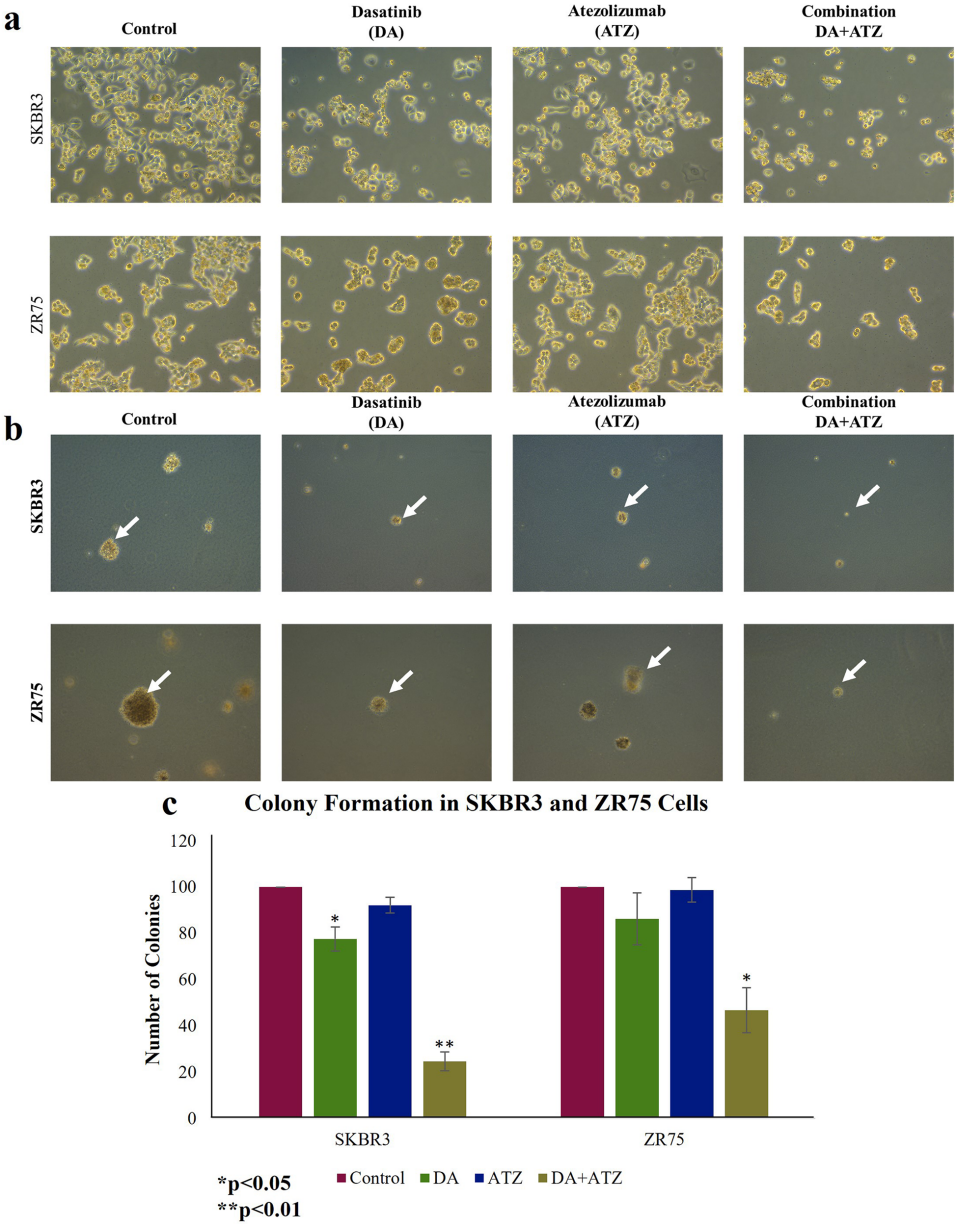
Effect of DA and ATZ on cell morphology of SKBR3 and ZR75, after 48 h (**a**). In controls, SKBR3 and ZR75 cells have round or polygonal morphology and form clusters. Upon treatment, we notice smaller clusters and cell death.(images a and b at ×20 magnification). The outcome of DA and ATZ on colony formation of SKBR3 and ZR75 cell lines in soft agar after 21 days (**b** & **c**). DA and ATZ inhibit colony formation of SKBR3 and ZR75 in comparison with their matched control cells. Colonies were counted manually and expressed as a percentage of treatment relative to the control (Mean ± SEM)

In order to validate the findings of this study, we attempted to block PD-L1 using the monoclonal antibody; Atezolizumab (ATZ). We revealed that ATZ monotreatment affects cancer cell morphology compared to untreated controls, as cells become slightly elongated and form smaller clusters. When DA and ATZ are combined, cell death incidents were observed. These results suggest similar outcomes when replacing BMS-202 with ATZ, which validates our findings (Fig. 9a). Further, the outcome of colony formation experiment suggests that ATZ can inhibit colony formation of SKBR3 in a significant manner (*p* < 0.05) compared to the control, but this was not observed in ZR75 cell line. When cells were treated with DA and ATZ combination, a significant reduction in colony formation was observed in SKBR3 (*p* < 0.01) and ZR75 (*p* < 0.05). Counting the resulting colonies revealed that the wells treated with DA and ATZ combination have the lowest number of colonies compared to monotreatment and control (Fig. 9b and c).

**Fig. 10:**
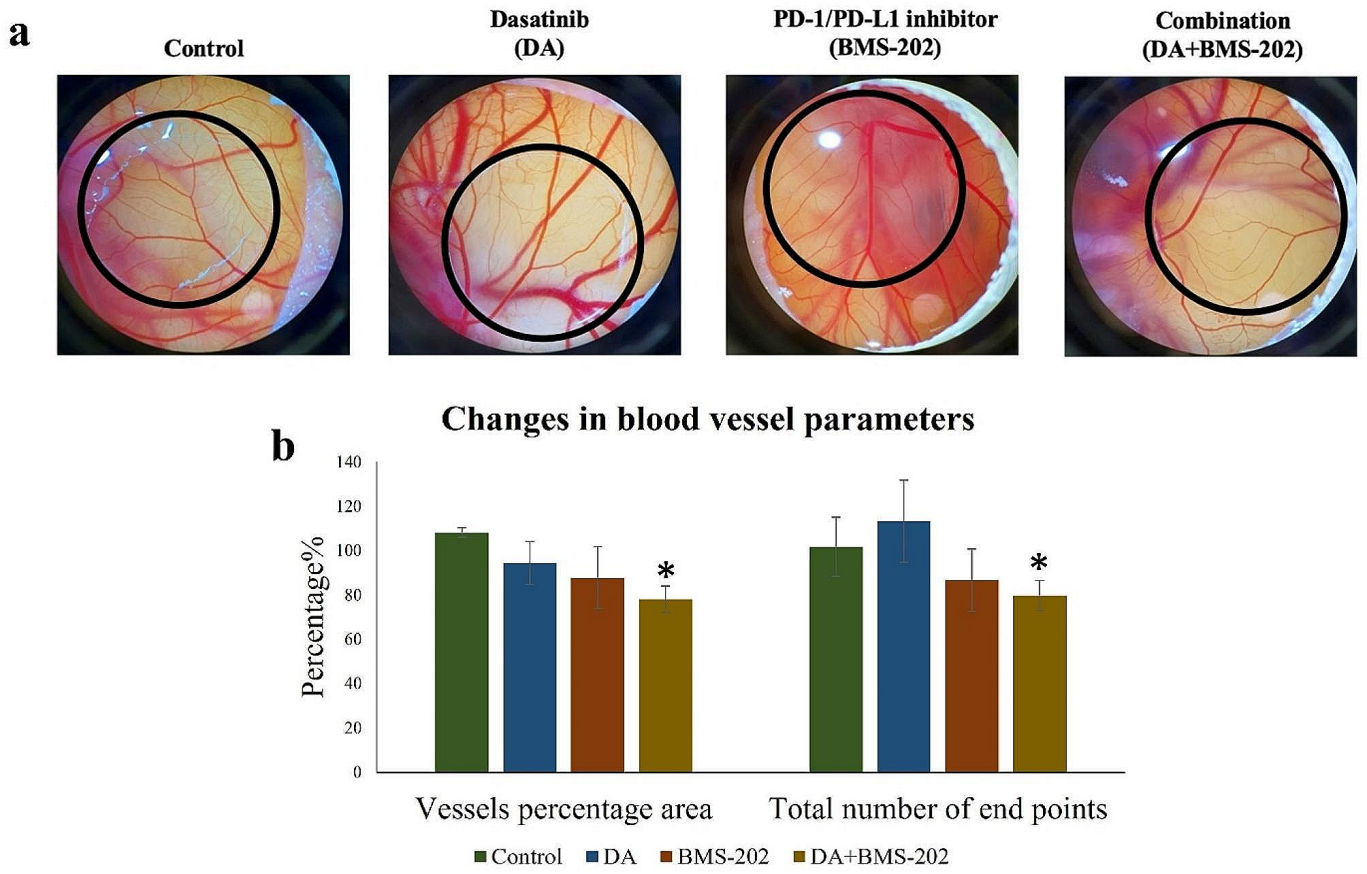
Effects of DA (1 µM) and BMS-202 (1 µM) inhibitors on angiogenesis using the CAM of the chicken embryo for 48 h. DA and BMS-202 in combination inhibit angiogenesis of the CAM compared with those exposed to DA and BMS-202, individually as well as with DMSO (control). CAM was treated for 48 h as shown in the materials and methods section (**a**). Images were quantified using AngioTool software to measure changes in blood vessel parameters (**b**)

Finally, based on the fact that angiogenesis plays a vital role in cancer progression, we investigated the effect of DA and BMS-202 on blood vessel development in-vivo using the CAM of chicken embryos. Herein, it is essential to highlight that a concentration of 1µM was selected based on the acceptable toxicity of these drugs on the embryo at 5 days of incubation. We found that the combination of DA and BMS-202 considerably inhibits blood vessel development of the CAM model compared to monotreatment of these inhibitors and their matched control (Fig. 10a). Quantification of blood vessel parameters revealed a significant decrease in vessel percentage area and the total number of endpoints in embryos treated with the combination therapy compared to monotreatment and controls (Fig. 10b).

## Discussion

In this study, we investigated the effects of DA and BMS-202 in HER2-positive human breast cancer cell lines (SKBR3 and ZR75) concerning cell viability, EMT event, cell invasion, colony formation, and angiogenesis in addition to their underlying molecular pathways. We herein report that DA and BMS-202 together significantly suppress cell viability of both cell lines, SKBR3 and ZR75, along with inhibition of colony formation. Moreover, our data show clearly that these inhibitors block normal blood vessel development of the CAM of chicken embryos.

Today, combination therapy approaches are emerging in the field of cancer management. DA was identified as a potential candidate for combination therapy in HER2-positive breast cancer by high-throughput screening [55]. The efficacy of DA as a part of combination therapy against HER2-positive breast cancer was shown in-vitro and in-vivo [35, 56–58]. On the other hand, BMS-202 was successfully combined with different treatment approaches for the treatment of HER2-positive breast cancer [59–62]. Also, we have previously shown that BMS-202 synergizes trastuzumab efficacy in suppressing HER2-positive breast cancer colony formation in-vitro [63]. To our knowledge, this is the first study to evaluate the outcome of DA and BMS-202 combination in HER2-positive breast cancer. From a clinical perspective, DA and BMS-202 combination therapy might result in decreasing DA dosage and hence minimize its side effects, such as cardiovascular toxicity and colitis [64, 65].

EMT is a critical event in cancer progression, where epithelial cells undergo molecular changes and promote the breakdown of intracellular tight junctions, loss of cell-cell contact, and epithelial cell features [66]. Also, cancer progression is associated with cell dedifferentiation along with loss of E-cadherin [67]. Moreover, various reports have shown loss and/or delocalization of E-cadherin and β-catenin expression patterns, in addition to enhanced expression of vimentin to trigger EMT, which further promotes cancer progression [66, 68, 69]. Additionally, there are other important key markers of EMT such as TGF-b, cytokeratin, α-smooth muscle actin (α-SMA), and fibroblast-specific protein-1 (FSP1) that have been identified earlier [66]. In this study, treatment with DA and BMS-202 enhanced the expression of E-cadherin and translocated (restored) it from the cytoplasm to the cell membrane to form a complex with β-catenin, while vimentin expression was downregulated. Accordingly, these drugs can prevent the EMT progression and therefore block cell invasion of the two HER2-positive breast cancer cell lines. These data are consistent with our previously published work regarding the outcome of Src/Abl inhibitor (SKI-606) on human cervical cancer cell lines, SiHa and HeLa [70]. However, the impact of our DA and BMS-202 combination on other important key markers of EMT should be investigated in future studies.

Moreover, we report that DA and BMS-202 inhibit colony formation of HER2-positive breast cancer cell lines, which could be considered an in-vivo tumor formation [71–74]. In order to validate our findings, we used another method of PD-L1 inhibition, which is the PD-L1 monoclonal antibody Atezolizumab (ATZ). We found that a combination of DA and ATZ can significantly inhibit the colony formation of SKBR3 and ZR75 as well. However, DA and BMS-202 combination was more effective in reducing the colony number in both cell lines, suggesting that BMS-202 might affect other downstream pathways related to cell growth and survival. Similar to our study, another investigation in breast cancer cell lines showed that DA induces cell cycle arrest, represses cell migration and invasion, and reduces colony formation *via* EGFR signaling [75]. In addition, in pancreatic cancer, DA induces E-cadherin/β-catenin expression while downregulating Slug in both in-vitro and in-vivo; thus, indicating a role of DA in reversing the EMT process [76]. DA also was shown to inhibit TGFβ-induced EMT in lung fibrosis and lung cancer [77, 78]. Similarly, the use of PD-1/PD-L1 inhibitors enhances the expression levels of E-cadherin and reduces N-cadherin expression, thus, inhibiting EMT in hepatocellular carcinoma cells [79]. Likewise, a study in thyroid cancer cells also demonstrated similar data, where PD-L1 was shown to repress E-cadherin expression, upregulate vimentin, and promote EMT [80]. We herein point out that DA and BMS-202 can affect these events in HER2-positive cell lines, which can indicate the potential use of such drugs for the treatment of highly invasive cancer types.

On the other hand, it is well known that angiogenesis plays a vital role in cancer progression; thus, inhibiting new blood vessel development is considered one of the major avenues in managing cancer metastasis [81, 82]. In this study, we explored the outcome of DA and BMS-202 individually and in combination on angiogenesis using one of the most common in-vivo models for this important event which is the CAM of the chicken embryo. Our data show clearly that DA and BMS-202 could significantly inhibit blood vessel development of the CAM. An earlier investigation of breast cancer showed that DA inhibits angiogenesis [75]. Likewise, Liang et al. (2008) showed that DA can inhibit angiogenesis in-vitro and in-vivo models [83]. The investigation showed that DA could block angiogenesis in HUVEC cells, chick aortic ring assay, and the human prostate cancer xenograft model [83]. In addition to cancer, DA has been shown to inhibit angiogenesis in rheumatoid arthritis [84], the mouse retina, and choroid [85]. Likewise, other studies have demonstrated a combination of PD-1/PD-L1 inhibitors with anti-angiogenesis drugs to inhibit angiogenesis and induce cancer immunity in lung, kidney, and liver carcinomas [86–90].

Regarding the molecular pathways induced or inhibited by the action of DA and BMS-202 on our cell line models, we found that treatment with a combination of DA and BMS-202 can inactivate HER2 receptor and deregulate the expression patterns of PI3K/AKT and β-catenin. It has been reported that oncogenic activation of the HER2 receptor triggers its downstream signaling pathways, including PI3K/AKT/mTOR and β-catenin, both of which are actively involved in cell metabolism, proliferation, invasion, migration, angiogenesis, apoptosis, and chemoresistance [50, 91]. Furthermore, in HER2-positive breast cancer, the role of AKT in enhancing JNK activation and its correlation with HER2 have been previously reported [92]. Consistent with our findings, previous studies have shown that DA blocks HER2 and AKT phosphorylation while targeting Src, and deregulating its phosphorylation in different cell line models [56, 57, 75, 93]. On the other hand, BMS-202 impedes the activity of the PD-1/PD-L1 axis by blocking PD-1 binding to its ligand, and therefore inhibiting their downstream proteins, such as AKT [63, 94]. Altogether, DA, and BMS-202 combination can significantly reduce cell viability, cell invasion, and colony formation in addition to angiogenesis inhibition via affecting key proteins such as HER2, AKT, JNK1/2/3, and β-catenin.Throughout this study, we noticed that SKBR3 cell line is more sensitive to treatment with DA and BMS-202 in comparison with ZR75. This is consistent with our findings regarding the higher expression of both Src and PD-L1 in SKBR3 compared to ZR75, similar to previous reports [25–27].

## Conclusions

This study reports for the first time the synergistic effects of dasatinib and PD-1/PD-L1 inhibitor (BMS-202) combination on HER2-positive breast cancer and its underlying mode of action. This investigation brings a novel therapeutic potential for these two drugs and their potent mechanism via PKI3/AKT and β-catenin pathways in HER2-positive cases. However, more studies, particularly in-vivo, preclinical, and clinical, are necessary to validate the safety and effectiveness of such a combination in cancer patients. Taken together, our combination might open new avenues for the management of HER2-positive breast cancers.

## Data Availability

The data presented in this study are contained within the article or supplementary material.

## Abbreviations

HER2: human epidermal growth factor receptor
PD-L1: programmed death ligand 1
DA: Dasatinib
EMT: epithelial-mesenchymal transition
CAM: chorioallantoic membrane
FACS: Fluorescence-activated cell sorting
